# Risks of forgotten double-J ureteric stents after ureterorenoscopic lithotripsy in Taiwan: a nationwide population-based study

**DOI:** 10.1038/s41598-020-77771-y

**Published:** 2020-11-26

**Authors:** Weiming Cheng, Yi-Chun Chiu, Yu-Hua Fan, Shu-Yi Lin, Sheng-Wen Chen

**Affiliations:** 1 Division of Urology, Department of Surgery, Urological Center, Taipei City Hospital, Zhongxiao Branch, No.87, Tongde Road, Nangang District, Taipei, 11556 Taiwan; 2 Division of Urology, Department of Surgery, Heping Fuyou Branch, Taipei City Hospital, Taipei, Taiwan; 3 Department of Urology, Taipei Veterans General Hospital, Taipei, Taiwan; 4 Department of Education and Research, Taipei City Hospital, Taipei, Taiwan; 5 Department of Urology, School of Medicine, National Yang-Ming University, Taipei, Taiwan; 6 Department of Exercise and Health Sciences, University of Taipei, Taipei, Taiwan

**Keywords:** Medical research, Urology

## Abstract

Delayed double-J ureteric stent (DJ) removal may cause severe morbidity. We aimed to identify high-risk patients for forgotten DJs to prevent iatrogenic injury and improve safety. Data of patients with DJs placed after ureterorenoscopic lithotripsy (URSL) between 2000 and 2013 from the National Health Insurance Database in Taiwan were included. Forgotten DJs were defined as indwelling DJs for > 6 months after URSL, which is approximately two times longer than the expiratory duration. Age at stenting, sex, socioeconomic status, specialty of stenting physician, comorbidities, postoperative emergency room visiting and abdominal plain x-ray filming frequencies, and alpha blocker use for > 7 days after stenting were analysed. Of 13,058 patients, 12,969 (99.31%) had timely removed DJs while 89 (0.68%) had forgotten DJs. Per a univariate analysis, patients with forgotten DJs were older, female, and of low socioeconomic status, and the use of more than one DJ for one URSL, placement by non-urologists, and less frequent postoperative abdominal plain x-ray filming and postoperative alpha blocker use were risk factors. Per a multivariate analysis, elderly patients (Odds ratio [OR] = 3.37, 95% confidence interval (CI) 1.36–8.32, p = 0.0085), DJ placement by non-urologists (OR = 9.63, 95% CI 6.09–15.24, p < 0.0001), more than two DJs for one URSL (OR = 2.93, 95% CI 1.58–5.42, p = 0.0006), and less frequent postoperative abdominal plain x-ray filming (OR = 0.66, 95% CI 0.51–0.86, p = 0.0016) were significantly associated with forgotten DJs. Forgotten URSL-related DJs are infrequent in Taiwan. Old age, complicated DJ insertion requiring more than two stents for one URSL, and stent placement by non-urologists are risk factors. Physicians should be aware of these high-risk patients.

## Introduction

Double-J ureteric stent (DJ) indwelling is one of the most common procedures in urology. DJs are placed electively prior to abdominal or pelvic surgeries in order to more effectively identify ureters, as well as after endourological procedures in order to prevent iatrogenic complications^1^. They are also used as a palliative treatment for obstructive uropathy resulting from ureteral stenosis or neoplasms. However, there are several adverse effects, such as lower urinary tract symptoms, flank pain, and haematuria, which are associated with this medical device. One of the most devastating complications is the unexpectedly delayed-removal of DJs, also known as forgotten DJs^2,3^. Urinary tract infection and encrustations are common symptoms related to forgotten DJs, and occasionally atrophy develops in the affected kidney due to long-term in-stent obstruction^4,5^. Since the management of forgotten DJs is challenging and requires additional endoscopic or even open procedures, there are studies that have focused on identifying the risk factors associated with forgotten DJs^2,6^. However, hospital-based studies could underestimate the incidence because patients may not always have their forgotten DJs placed and removed in the same institutes. Therefore, in the present study, we aimed to evaluate the possible risk factors for forgotten DJs in Taiwan using data derived from a nationwide population-based database.

## Materials and methods

The National Health Insurance (NHI) program was launched in March 1995. It is mandatory for the residents of Taiwan to join the NHI, and over 99.9% of the residents are enrolled in this program. The NHI research database (NHIRD), containing registration files and data corresponding to original claims for reimbursement, was integrated and maintained for research purposes. Medical data, including data regarding patient diagnoses and related ambulatory and hospitalization orders, procedures, and medications, can be obtained from this database. For privacy protection, data that could be used to identify individual patients or care providers were scrambled. All the researchers were requested to sign a written consent document declaring that they have no intention of obtaining information that may invade the privacy of patients or care providers.

We included claims from the NHIRD related to the placement of DJs after ureterorenoscopic lithotripsy (URSL) in adult patients between 2000 and 2013. The acceptable stent life of all these indwelling DJs within the human body is 3 months. Therefore, as a strict definition, we defined patients who did not undergo endoscopic, laparoscopic, or open surgical procedures that involved entry into the urinary tracts within 6 months after DJ placement as having forgotten DJs^7^. If the patients had more than two DJs, the performance of one removal procedure within 6 months would exclude them from the group with forgotten DJs. The following factors were compared between the groups with and without forgotten DJs: demographic characteristics, including sex and age at DJ indwelling; the presence of comorbidities, such as dementia and hemiplegia, which could affect a patient’s cognitive or self-care functions; socioeconomic status based on their monthly wages recorded in the NHI database^8^; and the specific type of specialist (urologists or non-urologists) under whose service the DJs were placed. Additionally, the frequency of postoperative emergency room visits, abdominal plain x-ray filming, and postoperative prescriptions of alpha blockers for a course of more than a week within 3 months after the DJ indwelling procedure were also compared. We hypothesized that less severe ureteric stent-related symptoms would result in the patients ignoring and forgetting about the presence of DJs inside their bodies, especially in cases in which the DJs were newly indwelled. Alpha blockers are reported to improve ureteric stent-related symptoms^9^; therefore, we tested the association of postoperative prescriptions of alpha blockers with forgotten DJs. A T test, Fisher’s exact test, and Pearson’s chi square test were applied for statistical analysis. A multivariate analysis was additionally performed to identify the factors associated with forgotten DJs. Further, *p* values of less than 0.05 were defined as statistically significant.

## Results

There were a total of 13,058 patients who underwent DJ indwelling from 2000 to 2013. Most patients (94.62%) received only one DJ for one URSL procedure while others received more than two DJs for one URSL procedure, which may indicate bilateral DJ indwelling or failed DJ indwelling during the surgical procedure (Table 1). Further, 89 patients (0.68%) did not have their DJs removed within 6 months after URSL, and they were assigned to the forgotten DJs group (Table 2).

**Table 1:** Number of DJs used in one URSL procedure (within 3 months).

Number of DJs	Patient numbers	%
1	12,355	94.62
2	650	4.98
3	49	0.38
4	3	0.02
6	1	0.01
Total	13,058	100.00

DJs, double-J ureteric stents; URSL, ureterorenoscopic lithotripsy.

**Table 2:** Numbers of patients with timely and delayed removed DJs.

	Patient numbers	%
**Received only 1 DJ**		
Timely removed DJs	12,279	94.03
Forgotten DJs	76	0.58
**Received 2 to 6 DJs**		
Timely removed DJs	690	5.28
Forgotten DJs	13	0.10
Total	13,058	100.00

DJs, double-J ureteric stents.

Patients with forgotten DJs tended to be over 65 years of age (42.7% vs. 20.37%, p < 0.0001), female (59.55% vs. 36.06%, p < 0.0001), and of low socioeconomic status (monthly wage < USD 640, 40.45% vs. 24.18%, p < 0.0001) compared to those without DJs. The use of more than one DJ for one URSL procedure (14.61% vs. 5.32%, p < 0.0009), DJ indwelling under the service of non-urologists (65.17% vs. 11.48%, p < 0.0001), less frequent postoperative abdominal plain x-ray filming (0.49 ± 1.05 times vs. 1.20 ± 1.43 times, p < 0.0001), and less frequent postoperative prescriptions for alpha blockers to be taken for more than 7 days (91.01% vs. 83.20%, p = 0.0402) were also associated with the occurrence of forgotten DJs. The presence of comorbidities, including dementia or hemiplegia, were not found to be associated with forgotten DJs (Table 3).

**Table 3:** Univariate analysis of potential risk factors for forgotten DJs.

Risk factors	Timely-removed DJs (n = 12,969)		Forgotten DJs (n = 89)		P value^※^
	Number	%	Number	%	
	12,969		89		
**Sex**					** < 0.0001**
Female	4677	36.06	53	59.55	
Male	8292	63.94	36	40.45	
**Age (years)**					** < 0.0001**
20–64	10,327	79.63	51	57.30	
> 65	2642	20.37	38	42.70	
**Service specialty**					** < 0.0001**
Non-urology	1489	11.48	58	65.17	
Urology	11,480	88.52	31	34.83	
**ER visiting frequency, means ± SD**^**a**^	0.01 ± 0.08		0.02 ± 0.15		0.2855
**KUB filming frequency, means ± SD**^**a**^	1.20 ± 1.43		0.49 ± 1.05		** < 0.0001**
**Alpha blocker usage for over 1 week**					**0.0492**
No	10,790	83.20	81	91.01	
Yes	2179	16.80	8	8.99	
**Number of DJs placed**				0.00	**0.0009**
Received only 1 DJ	12,279	94.68	76	85.39	
Received 2 to 6 DJs	690	5.32	13	14.61	
**Comorbidities**					
Dementia					0.0799*
No	12,340	95.15	81	91.01	
Yes	629	4.85	8	8.99	
Hemiplegia or paraplegia					0.2523
No	12,061	93.00	80	89.89	*
Yes	908	7.00	9	10.11	
**Monthly wages**					** < 0.0001**
Well-defined monthly wage					
≥ USD 1280	1879	14.49	5	5.62	
USD 640–1279	3819	29.45	13	14.61	
< USD 640	3136	24.18	36	40.45	
Without a well-defined monthly wage					
Farmers and fishermen	2295	17.70	15	16.85	
Others (veterans, low-income individuals, and individuals enrolled in the NHI through local government offices)	1840	14.19	20	22.47	

Statically significant P values are given in bold.

DJs, double-J ureteric stents; OR, odds ratio; SD, standard deviation; ER, emergency room; KUB, kidney, ureter, and bladder; NHI, National Health Insurance.

^※^Pearson Chi-square test.

*Fisher's exact test.

^a^Two sample t tests.

Based on the results of the multivariate analysis, the older the patients, the higher the risk of forgotten DJs (odds ratio for patients between 40 and 64 years of age = 2.49, 95% confidence interval (CI) 1.05–5.90, p = 0.0384; odd ratios for patients older than 65 years of age = 3.37, 95% CI 1.36–8.32, p = 0.0085). The placement of DJs under the service of non-urologists (odds ratio = 9.63, 95% CI 6.09–15.24, p < 0.0001), use of more than two DJs for one URSL procedure (odds ratio = 2.93, 95% CI 1.58–5.42, p = 0.0006), and less frequent postoperative abdominal plain x-ray filming (odds ratio = 0.66, 95% CI 0.51–0.86, p = 0.0016) were also risk factors for forgotten DJs (Table 4). Sex, socioeconomic status, postoperative emergency room visits, and postoperative prescriptions of alpha blockers for a course of more than 7 days were not found to be associated with the occurrence of forgotten DJs based on the multivariate analysis results.

**Table 4:** Multivariate analysis of potential risk factors for forgotten DJs.

Risk factors	OR	95% CI	P value
**Sex**			
Female	1.00	–	–
Male	0.78	0.49–1.23	0.2861
**Age (years)**			
20–39	1.00	–	–
40–64	2.49	1.05–5.90	**0.0384**
> 65	3.37	1.36–8.32	**0.0085**
**Service specialty**			
Non-urology	9.63	6.09–15.24	** < 0.0001**
Urology	1.00	–	–
**KUB filming frequency**, **means ± SD**^**§**^	0.66	0.51–0.86	**0.0016**
**Alpha blocker usage for over 1 week**			
No	1.00	–	–
Yes	0.59	0.27–1.28	0.1799
**Number of DJs placed**			
Received only 1 DJ	1.00	–	–
Received 2 to 6 DJs	2.93	1.58–5.42	**0.0006**
**Monthly wages**			
Well-defined monthly wage			
≥ USD 1280	1.00	–	–
USD 640–1279	1.05	0.37–2.99	0.9208
< USD 640	2.22	0.84–5.92	0.1098
Without a well-defined monthly wage			
Farmers and fishermen	1.28	0.44–3.71	0.6469
Others (veterans, low-income individuals, and individuals enrolled in the NHI through local government offices)	2.31	0.84–6.37	0.1060

Statically significant P values are given in bold.

DJs, double-J ureteric stents; OR, odds ratio; SD, standard deviation; CI, confidence interval; KUB, kidney, ureter, and bladder; NHI, National Health Insurance.

^§^Means ± SD is not available in multivariate analysis

## Discussion

DJs which were first introduced in 1978 by Finney et al*.* are commonly used in urology procedures^10^. One of the most serious complications associated with this medical device is the unexpectedly delayed removal of DJs, which is also known as forgotten DJs. This complication could cause tremendous morbidity in patients as well as burden to the healthcare system. Sancaktutar et al*.* compared the treatment costs between forgotten and timely-removed DJs. The average treatment costs for patients with forgotten DJs are 6.9-fold higher than those associated with timely removal. In addition to the financial burden, the complications of forgotten DJs also impair patient quality of life and reduce the labour force^11^. Therefore, it is important to develop measures for preventing or decreasing the possibility of forgotten DJs.

In order to prevent the possibility of forgotten DJs, Hadaschik et al*.* investigated a novel biodegradable ureteric stent which can completely degrade within 7 to 10 weeks^12^. However, it is not suitable in the case of certain conditions, such as ureteral stricture, which may need longer DJ placement durations. It is also much more expensive than regular DJs and is not available worldwide. An electronic stent register (ESR) and a stent extraction reminder facility were developed by Lynch and Lin, respectively*.* A daily reminder email would automatically be sent to clinical staff if the maximal stent life was reached until the stent was removed, and the removal was updated on the ESR^13,14^. Similarly, Sancaktutar et al*.* used a multimedia message service to remind patients with DJs. None of the patients in the group that was messaged had their DJs forgotten. Nevertheless, it is labour intensive, and not every patient has electronic communication equipment. Reminder messages may fail to reach all the patients due to erroneous phone numbers or changed phone numbers^15^. The identification of the risk factors for forgotten DJs remains as a simple measure that could help in preoperative patient education and postoperative follow-up.

In the literature, the incidence of forgotten DJs ranges from 3.8 to 16%, which is higher than that in our present study^2,6,16–18^. There are several explanations for the differences. First, the definitions of forgotten DJs are different. For example, Lin et al*.* defined forgotten DJs as DJs indwelling between 3 and 12 months according to material type and manufacturer guidelines while Divakaruni and Tang used 6 months as the cut-off point^2,6,18^. Second, the inclusion criteria of patients were not consistent among different studies. In the present study, we only included patients who underwent DJ indwelling after URSL rather than those with other indications, such as ureteral stricture. Third, the accessibility of healthcare systems would have an influence on the incidence of forgotten DJs. Divakaruni concluded that the higher incidence in their study might be attributed to their patients’ lack of health insurance^6^. On the other hand, the NHI covers over 99.9% of the residents in Taiwan, which reduces the financial burden of medical care and may decrease the incidence of forgotten DJs in Taiwan.

In our present study, age was found to be an important risk factor for forgotten DJs, which is consistent with the results of other studies. Lin et al*.* found that patients older than 60 years of age were more likely to have forgotten DJs^2^. The non-compliance with medical advice in the elderly is believed to be unintentional and is related to a low educational status or cognitive and physical impairments^19^. Necessary help from healthcare providers and thorough perioperative education provided to their family members may improve the compliance of elderly patients in having their DJs removed on time.

The indwelling of DJs under the service of non-urologists is greatly associated with forgotten DJs after URSL. Compatible with our results, Lin et al. found that all the forgotten DJs in their auto-registration monitoring system were inserted under the service of non-urologists^14^. Patient education in relation to informational needs and postoperative complications may play an important role in this aspect^20,21^. Physicians other than urologists may not be familiar with this medical device and do not emphasize on the necessity of removing DJs in time. On the other hand, when patients are treated by urologists, more thorough counselling, and education concerning the symptoms and complications associated with DJs would be provided. Moreover, postoperative outpatient appointments with urologists, rather than with other specialists, would also result in the opportunity to review the DJs present inside the patients’ bodies and to remove them.

Similarly, more frequent postoperative abdominal plain x-ray filming within 3 months was associated with a lower incidence of forgotten DJs in our present study. One possible reason for this relationship may be because the larger the number of complaints regarding ureteric stent-related symptoms, the more frequently postoperative abdominal plain x-ray filming is performed. Ureteric stents are evident on abdominal plain x-ray films and can serve to remind physicians, including urologists and non-urologists, that there are stents awaiting removal inside the patients’ bodies.

Patients with more than two DJs for a single session of URSL tended to have forgotten DJs in our study. There are several conditions, such as bilateral ureteral stones and failed DJ insertion during surgery, which are associated with the application of more than two DJs for one URSL procedure. Ureteric stents tended be placed for longer periods in such conditions, and the urinary system would get desensitized to the irritation caused by the DJs over time^22^. Consequently, patients would become more tolerant to ureteric stent-related symptoms, and therefore, a higher associated incidence of forgotten DJs would arise.

There are several limitations of the present study. First, to identify patients effectively with unforgotten DJs is impossible since there is no corresponding International Classification of Disease (ICD) code for this medical condition. We defined patients with DJs as those registered with such a claim after USRL in the NHIRD. However, this does not always guarantee that the DJ is effectively present within the patient’s body. We also applied a stricter definition for forgotten DJs. We excluded all patients who received procedures that involved entry into their urinary tracts after URSL. We thought that there was a chance for the urologist to notice the presence of the DJ, and then to remove it on time even if everyone forgot it. In some rare circumstances, the string attached to the ureteric stent will be kept in place while the stent will be removed manually. However, this is not routine clinical practice, and some urologists would still claim the removal procedure from the NHI. Second, the NHIRD contains only registration profiles and claim-related data for reimbursement. Therefore, it was not possible to include the results of laboratory examinations and reports of imaging examinations for further analysis in this study. Third, ureteric stent-related symptoms play an important role in the occurrence of forgotten DJs. However, there are no records of symptoms and signs in the NHIRD. Consequently, it was not possible to classify patients based on the severity of the ureteric stent-related symptoms in the present study, and we could only use indirect evidence, such as the frequency of abdominal plain x-ray filming, as risk factors. Fourth, the identification of comorbidities in the present study was based on diagnostic codes that are relatively inaccurate. The severity of the comorbidities that contribute to the incidence of forgotten DJs is also not recorded in the NHIRD. Fifth, the exact reasons for the application of more than two DJs for one URSL procedure cannot be identified from the data in the NHIRD.

Nevertheless, there are advantages of the present study. To the best of our knowledge, this is the first nationwide study involving a large sample size which was performed to recognize the possible risk factors for forgotten DJs across difference institutes. The findings of the present study could be used for risk stratification in patients with DJs. Different measures could be implemented for patients with varied risks in order to save human resource costs. For example, application of shared decision-making with patients who have urolithiasis, extracorporeal shock wave lithotripsy rather than URSL for patients at risk of forgotten DJs, and emphasizing avoidance of DJ placement in uncomplicated URSL cases. Other measures could be implementing an auto-registration monitoring system of DJs, and use of an online interactive communication application for education and follow-up of patients at risk of forgotten DJs. All these could help to improve patients’ awareness and compliance.

## Conclusions

Forgotten DJs are an infrequent complication of stent indwelling after URSL procedures in Taiwan. Old age, complicated DJ insertion requiring more than two stents for one URSL session, and stent placement under the service of non-urologists are risk factors. Physicians should remain alert on encountering these high-risk patients, and detailed preoperative education is warranted. Frequent postoperative follow-up sessions with abdominal plain x-ray filming are also important for detecting the presence of DJs and should be arranged for every patient after DJ indwelling procedures.

## Data Availability

Data utilized in our study are available from the National Health Insurance Research Database (NHIRD) published by Taiwan National Health Insurance (NHI) Bureau. Based on the “Personal Information Protection Act” in Taiwan, data cannot be made publicly available due to legal restrictions imposed by the government of Taiwan. Requests for data can be sent as a formal proposal to the NHIRD (http://nhird.nhri.org.tw).
